# Preadmission medications and recent falls in older inpatients: an observational study

**DOI:** 10.1007/s11096-024-01859-y

**Published:** 2025-02-07

**Authors:** Louise Clarkson, Anthony Griffiths, Shu-Kay Ng, Alfred K. Lam, Tien K. Khoo

**Affiliations:** 1 School of Medicine & Dentistry, Griffith University, Gold Coast, QLD Australia; 2 Northern New South Wales Local Health District, Lismore, NSW Australia; 3 Graduate School of Medicine, University of Wollongong, Wollongong, NSW Australia

**Keywords:** Anticholinergic effect on cognition score, Drug burden index, Fall, Medication regimen complexity index, Preadmission polypharmacy

## Abstract

**Background:**

Falls in older adults might increase due to polypharmacy.

**Aim:**

This study aimed to explore the association between preadmission medications and history of falls in older inpatients.

**Method:**

This observational study of inpatients aged ≥ 65 years was conducted over 4 years at Ballina Hospital, Australia. The Medication Regimen Complexity Index (MRCI), Drug Burden Index (DBI), and Anticholinergic Effect on Cognition (AEC) scores were calculated for preadmission medications. Polypharmacy and falls questionnaires were administered to identify falls in the past 6 months and aptitude toward medication use.

**Results:**

Overall, 194 participants with a mean age of 80.2 (SD 8.0) years were included. The mean daily number of regular medications was 7.8 (SD 3.9) and the mean MRCI score was 22 (SD 12.6). Among the participants, 107 (55%) reported falls in the past 6 months and 47 (24%) reported ≥ 2 falls. Age and hearing impairment were positively associated with falls (*p* = 0.007 and *p* = 0.003, respectively). History of falls was positively associated with a MRCI score of ≥ 20 (*p* = 0.018), an AEC score of ≥ 2 (*p* = 0.010) and a DBI score of ≥ 1 after adjustment for age (*p* = 0.041). Forgetting medications was associated with falls (*p* = 0.043). Antihypertensive use did not increase falls risk.

**Conclusion:**

Implementing a decisive approach to simplify complex medication regimens, along with patient-focused medication management strategies, may help reduce the risk of falls in older adults. Sedatives and anticholinergic medications increase the risk of falls and should be avoided whenever possible.

**Supplementary Information:**

The online version contains supplementary material available at 10.1007/s11096-024-01859-y.

## Impact statements

- The study highlights the necessity of regular medication reviews for older patients to identify medications that may increase the risk of falls. This includes minimizing/avoiding sedatives and anticholinergic agents.
- Findings indicate that a highly complex medication regimen and forgetting to take medications are associated with an increased risk of falls. This highlights the importance of simplifying medication regimens and implementing effective medication management systems to enhance compliance.
- The study also adds to existing evidence that increasing age and hearing impairment are significant risk factors for falls. Targeting fall prevention strategies toward these patients may be beneficial.

## Introduction

Falls are common among older adults and pose a substantial risk of physical injuries. Approximately 30% of older adults aged 65 years or older are expected to experience at least one fall annually [1]. Approximately 10% of falls cause serious injuries, such as fractures or head trauma [2]. Additionally, older adults with a previous history of falls have an increased risk of experiencing another fall [3].

Polypharmacy (using 5 or more medications) has long been established as an independent risk factor for falls among older patients [4–6]. Therefore, managing complex medication regimens in potentially vulnerable and multimorbid patients is challenging. Reduced medication adherence has been suggested as a mechanism by which polypharmacy can increase the risk of falls [7]. As polypharmacy increases, the risk of being prescribed a medication that may increase the risk of falls will also increase. Thus, the presence of particular “fall risk-increasing drugs” may be more important than the number of medications [8, 9]. Medications, including diuretics, antihypertensives, proton pump inhibitors, sedatives, antidepressants, and antipsychotics, have been suggested to increase the risk of falls [10–14]. However, the results have been inconsistent between studies, and further investigation is needed. Anticholinergic and sedative medications have known adverse effects that include confusion, drowsiness and blurred vision. The use of these medications has been associated with a reduction in balance and increased risk of falls in several studies [15–18] The Drug Burden Index (DBI) is a tool used to measure cumulative exposure to anticholinergic and sedative medications [19]. Previous research has indicated that the DBI score may be positively associated with an increased risk of falls [20–22].

### Aim

This study aimed to explore the association between falls, medications, and aptitude toward medication use in older acute medical inpatients by examining their preadmission medications and recent history of falls. Specifically, associations between fall history and the number of medications, complexity of medications, compliance with medication, and use of anticholinergic or sedative medications were examined. Furthermore, other common medication types, such as antihypertensives, were examined to identify associations.

### Ethics approval

This study was approved by the Northern New South Wales Human Research Ethics Committee (Reference: 2018/ETH00453).

## Method

### Participants

This was an observational study conducted over 4 years from March 2019 to March 2023 at Ballina Hospital. A total of 194 inpatients aged ≥ 65 years participated in this study. The inclusion criteria included individuals who were community-dwelling before hospital admission. Participants were eligible regardless of the reason for their hospital admission. The exclusion criteria included individuals with insufficient knowledge of English (defined as insufficient to perform the cognitive tests and questionnaires in the opinion of the assessor), those with delirium determined by a 4AT score of 4 or more, and those with a documented history of dementia or significant cognitive impairment determined by a score of < 21 on the Standardized Mini-Mental State Examination (SMMSE) [23, 24]. Informed consent was obtained from all participants.

### Assessments

Participants were first screened using the 4AT delirium assessment tool [24]. Furthermore, cognition was assessed using the SMMSE and Montreal Cognitive Assessment (MOCA) [23, 25]. A MOCA score of ≤ 25/30 indicated mild cognitive impairment. Core baseline medical data, including reasons for admission, comorbidities and documented falls in the past 6 months, were collected from the hospital’s electronic medical records. The admission medication list was obtained from the medication management plan completed by the hospital pharmacist on admission. As part of routine clinical practice this includes a patient interview, a review of any patient’s medications brought to the hospital, dispensing history and a GP summary where available.

A fall was defined as “an unexpected event in which the person comes to rest on the ground, floor, or lower level” [26]. Participants were asked about the number of falls they had experienced in the past 6 months. A positive history of fall was documented where there was either a self-reported fall or a documented fall in the hospital's electronic medical records.

Participants completed a questionnaire on variables of interest, including the patient’s perception of prescribed medication, medication compliance, and subjective sensory impairments, such as hearing and visual impairments (see Supplementary material).

The participants’ regular exposure to medications with anticholinergic or sedative properties was quantitatively evaluated using the DBI. The linear additive model quantified the cumulative anticholinergic and sedative load to an individual using the following formula:

$${\text{DBI }} = \Sigma {\text{D}}/\left( {{\text{D }} + \, \partial } \right)$$

where D indicates the daily dose of medication taken by the individual and ∂ is the minimum recommended dose of the drug. The total drug burden was calculated by summing the scores of each anticholinergic or sedative medication. Drugs that have both effects were classified as anticholinergics [27]. The DBI was treated as a binary variable. A DBI score of “0” indicated that the participant was not taking anticholinergic or sedative medications. The DBI was classified as ‘high’ when scores were ≥ 1. These approaches have been previously used [28]. Regular medications were examined, and medications with potential anticholinergic or sedative effects and their minimum effective daily dose were identified using data from a large published study [29].

The cumulative anticholinergic cognitive activity of all medications was estimated using the Anticholinergic Effect on Cognition (AEC) score. The AEC scale follows principles similar to those of the well-known Anticholinergic Cognitive Burden (ACB) scale [30]. Medications are assigned a score of 1–3 by an expert panel, depending on their anticholinergic activity [31]. Despite being less used globally, the AEC score is advantageous for this study because the medications included are widely used in Australia compared with those used in the ACB scale.

The complexity of a participant’s drug therapy was quantified using the Medication Regimen Complexity Index (MRCI) [32]. The MRCI is a 65-item tool that evaluates the “medication load” beyond just the number of medications and considers the complexity of the drugs taken. It consists of three subscores: dosage forms, dosing frequency, and additional dosing instructions, which are summed to form a single patient MRCI score. The MRCI is a valid tool for assessing medication regimen complexity and may indicate patients at risk of nonadherence [33]. The stratification of MRCI scores varies between studies. Previous studies have defined MRCI scores from > 15 to > 25 as “high” [34–37]. In this study, an MRCI cutoff point of ≥ 20 was chosen based on previous cut-off values used to dichotomize the cohort into two groups based on medication complexity. A MCRI of below 20 was classed as ‘low’ complexity and ≥ 20 ‘high’ complexity.

Medications were categorized based on the Australian Medicines Handbook. Antihypertensive agents were categorized into thiazides and related diuretics, angiotensin-converting enzyme inhibitors (ACEIs), angiotensin receptor antagonist blockers (ARBs), calcium channel blockers (CCBs), beta blockers (BBs), and other antihypertensives.

### Statistical analysis

Data are presented as frequency (percentage) for binary or categorical variables and mean (standard deviation [SD]) for continuous variables. Continuous variables were checked for normal distribution using the Kolmogorov–Smirnov and Shapiro–Wilk tests. Falls were classified as binary (any number of falls vs. no falls). Participants’ demographic and medication-related characteristics were compared between the no falls and falls groups using chi-squared test for categorical variables and independent-samples t-test for continuous variables. Binary logistic regression was then used to assess for association between medication related variables and history of falls, with adjustment for age. The variables examined included number of medications taken, medication types, DBI score ≥ 1, AEC score ≥ 2, MRCI score ≥ 20 and medication-taking behaviors. Statistical analyses were performed using SPSS Statistics Version 29 for Windows (SPSS Inc., Chicago, IL, USA).

## Results

A total of 194 participants were included in this study with a mean age of 80.2 years (SD 8.0), 108 (55.7%) were female. Additionally, 42% were assessed as having mild cognitive impairment, 50% reported having vision issues, and 50% reported having hearing impairment. The prevalence of preadmission polypharmacy was high (mean number of total medications = 9.5 (SD 4.6) and regular medications = 8.0 (SD 3.9)) (Table 1).

**Table 1:** Characteristics of the participants

	All participants (*n* = 194)	Participants with no falls (*n* = 87, 44.8%)	Participants with one or more falls (*n* = 107, 55.2%)	*p*-value
Age at assessment, years	80.2 (8.0, 65.3–102.7)	78.5 (8.0, 65.3–95.5)	81.6 (7.7, 67.0–102.7)	***p* = 0.007**
Gender *Female Male*	108 (55.7%) 86 (44.3%)	43 (49.4%) 44 (50.6%)	65 (60.7%) 42 (39.3%)	*p* = 0.114
Mild cognitive impairment *No Yes*	81 (41.8%) 112 (57.7%)	38 (43.7%) 49 (56.3%)	44 (41.1%) 63 (58.9%)	*p* = 0.790
Problems with vision (self-reported) *No Yes*	96 (49.5%) 98 (50.5%)	45 (51.7%) 42 (48.3%)	51 (47.7%) 56 (52.3%)	*p* = 0.574
Hearing impairment (self-reported) *No Yes*	97 (50.3%) 96 (49.7%)	54 (62.1%) 33 (37.9%)	44 (41.1%) 63 (58.9%)	***p* = 0.003**
Total number of medications	9.5 (4.6, 2–26)	9.2 (5.0, 2–24)	9.8 (4.2, 2–26)	*p* = 0.139
Number of regular medications	8.0 (3.9, 1–21)	7.8 (4.1, 1–19)	8.1 (3.7, 1–21)	*p* = 0.331
Number of regular medications ≥ 10	57 (29.4%)	22 (25.3%)	35 (32.7%)	*p* = 0.259
MRCI	21.96 (12.6, 4–69)	21.1 (13.7, 4–69)	22.6 (11.5, 5–69)	*p* = 0.083
MRCI ≥ 20	93 (47.9%)	33 (37.9%)	60 (56.1%)	***p* = 0.012**

Values are presented as mean (SD, range) or frequency (%)

A total of 107 (55%) participants had experienced at least one fall in the past 6 months (patient reported or documented in their hospital notes). In 59% of these participants (*n* = 63), a fall was a part of the reason for hospital admission. Among the participants, 47 (24.2%) reported ≥ 2 falls.

From Table 1, participants with falls were older (mean age 81.6) compared to those without falls (mean age 78.5) (*p* = 0.007). Furthermore, patients with a history of falls were also more likely to report hearing impairment (58.9% versus 37.9%, *p* = 0.003).

An increase in the number of medications was not significantly associated with experiencing a fall. However, having a high complexity of medication (MRCI score ≥ 20) was positively associated with experiencing ≥ 1 fall in the past 6 months (*p* = 0.012). This further increased in significance once adjusted for age (adjusted odds ratio (AOR) = 2.0, 95% confidence interval (CI): 1.1–3.7, *p* = 0.018).

**Fig. 1:**
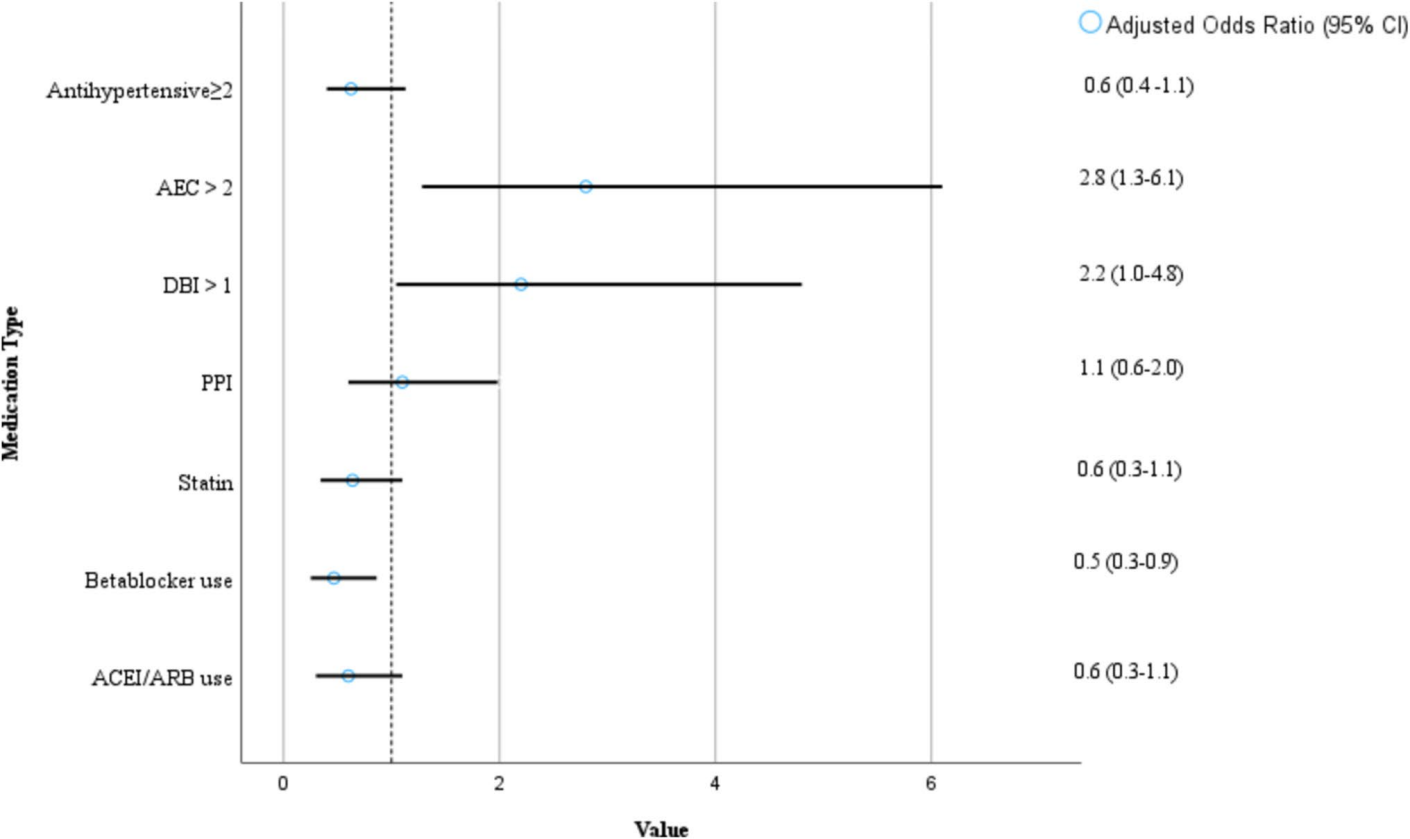
Pharmacological treatment type and history of falls in the past 6 months adjusted for age. The dotted vertical line indicates an adjusted odds ratio of 1 (no difference) AEC = Anticholinergic Effect on Cognition Score, DBI = Drug Burden Index Score, PPI = Proton Pump Inhibitor, ACEI = Angiotensin Converting Enzyme Inhibitor, ARB = Angiotensin Receptor Blocker

Table 2 lists the different medication groups examined and their prevalence of use between fallers and nonfallers. A high use of anticholinergic medications as indicated by an AEC score ≥ 2 was significant for a history of falls (*p* = 0.034). As illustrated in Fig. 1, both a DBI score ≥ 1 or an AEC score ≥ 2 was positively associated with experiencing ≥ 1 fall when adjusted for age (AOR = 2.2, 95% CI: 1.0–4.8, *p* = 0.041 and AOR = 2.8, 95% CI: 1.3–6.1, *p* = 0.010, respectively). Taking ≥ 2 antihypertensives was not associated with a history of falls. Closer analysis was limited by the sample size, with only ACEIs/ARBs and BBs being taken in sufficient numbers. BB use was negatively associated with a history of falls (*p* = 0.039).

**Table 2:** Pharmacological treatment on admission

	All participants (*n* = 194)	Participants with no falls (*n* = 87, 44.8%)	Participants with one or more falls (*n* = 107, 55.2%)	*p*-value
DBI = 0	100 (51.5%)	50 (57.5%)	50 (46.7%)	*p* = 0.136
DBI ≥ 1	40 (20.6%)	14 (16.1%)	26 (24.3%)	*p* = 0.160
AEC ≥ 2	40 (20.6%)	12 (13.8%)	28 (26.2%)	***p* = 0.034**
Antihypertensive Medication				
Thiazides and related antihypertensives	19 (9.8%)	10 (11.5%)	9 (8.4%)	*p* = 0.472
ACEIs	51 (26.3%)	26 (29.9%)	25 (23.4%)	*p* = 0.305
ARBS	55 (28.4%)	28 (32.2%)	27 (25.2%)	*p* = 0.285
CCBs	43 (22.2%)	21 (24.1%)	22 (20.6%)	*p* = 0.551
BBs	78 (40.2%)	42 (48.3%)	36 (33.6%)	***p* = 0.039**
Other antihypertensives	16 (8.2%)	8 (9.2%)	8 (7.5%)	*p* = 0.665
Statin Use	104 (53.6%)	53 (60.9%)	51 (47.7%)	*p* = 0.066
Proton Pump Inhibitor Use	86 (44.3%)	36 (41.4%)	50 (46.7%)	*p* = 0.456
Anticoagulant Use	51 (26.3%)	23 (26.4%)	28 (26.2%)	*p* = 0.966
Antiplatelet Use	74 (38.1%)	38 (43.7%)	36 (33.6%)	*p* = 0.152

ACEI = Angiotensin-Converting Enzyme Inhibitor, ARB = Angiotensin Receptor Blocker**,** CCB = Calcium Channel Blocker, BB = Betablockers

**Fig. 2:**
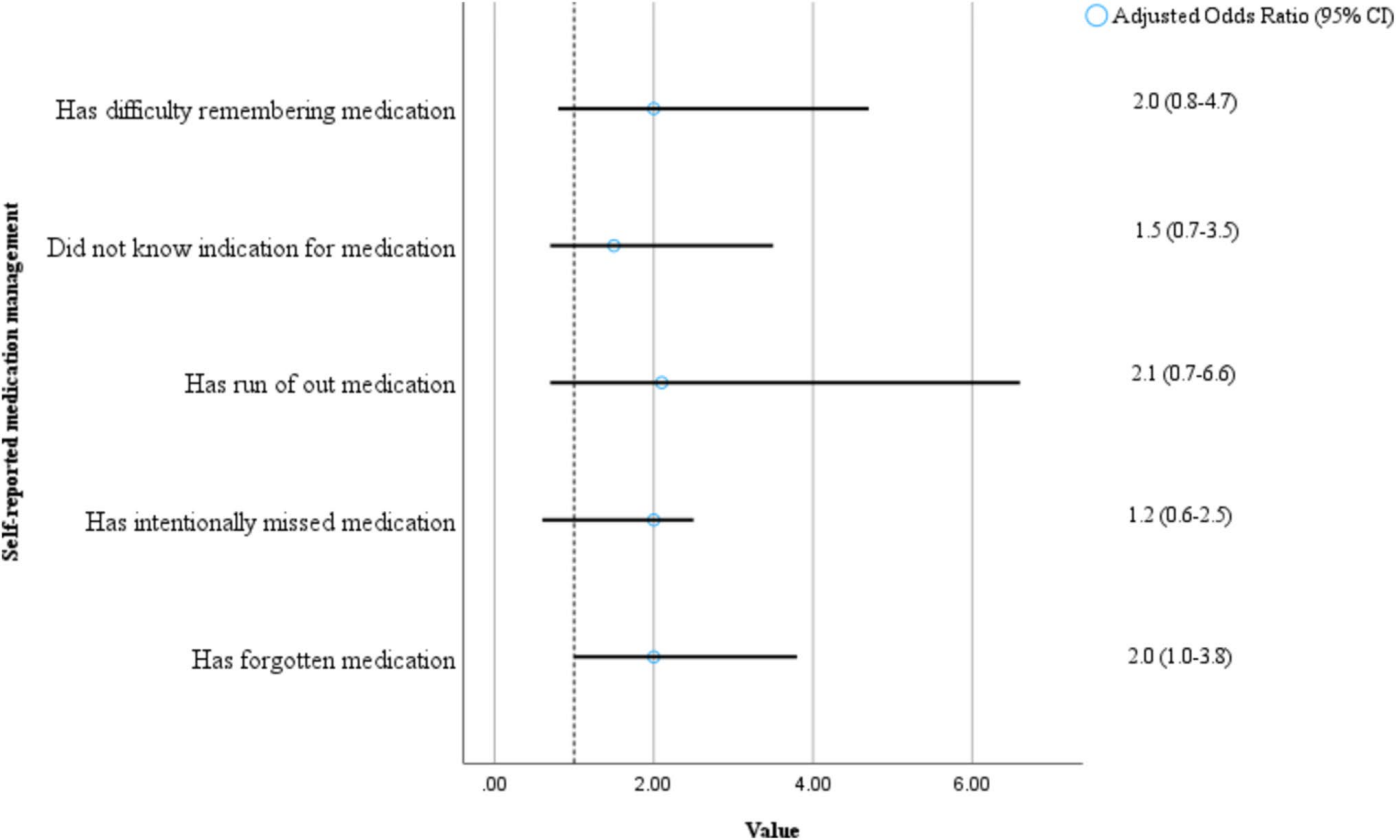
Medication management and history of one or more falls. Adjusted for age

Among the participants, 89% were taking their medications independently (without help) before admission. Of the 194 participants, 31 (16.5%) reported that they did not know why they were taking their medications, 59 (30.4%) reported that they could forget their medication, and 82 (42.3%) had an indicator of reduced compliance (either forgetting medication, running out of medication, or intentionally missing medication) (Table 3). Participants who reported forgetting medications were found to have a positive association with ≥ 1 fall in the past 6 months (*p* = 0.043) (Fig. 2). Having a hearing impairment, problems with vision or mild cognitive impairment was positively associated with patients not understanding why they were taking medication (*p* = 0.019, *p* = 0.024 and *p* = 0.012 respectively). Having a hearing impairment or problems with vision was associated with patients self-reporting difficulty remembering medication (*p* = 0.016 and *p* = 0.020).

**Table 3:** Self-reported medication-taking behaviors

	All patients Total (*n* = 194)
I know why I am taking medication	
*Strongly agree*	73 (37.6%)
*Agree*	70 (36.1%)
*Undecided*	19 (9.8%)
*Disagree*	23 (11.9%)
*Strongly disagree*	9 (4.6%)
I have no difficulties remembering to take my medication	
*Strongly agree*	100 (51.5%)
*Agree*	52 (26.8%)
*Undecided*	12 (6.2%)
*Disagree*	22 (11.3%)
*Strongly disagree*	8 (4.1%)
Do you ever forget your medication?	
*Yes*	59 (30.4%)
*No*	135 (69.6%)
I never run out of medication	
*Strongly agree*	119 (61.3%)
*Agree*	55 (28.4%)
*Undecided*	5 (2.6%)
*Disagree*	14 (7.2%)
*Strongly disagree*	1 (0.5%)
At times, I intentionally do not take some of my medication	
*Strongly agree*	13 (6.7%)
*Agree*	24 (12.4%)
*Undecided*	9 (4.6%)
*Disagree*	31 (16.0%)
*Strongly disagree*	117 (60.3%)
Someone else helps me with my medication	
*Yes*	22 (11.3%)
*No*	172 (88.7%)
Dose administration aid packed by the pharmacy	
*Yes*	62 (32%)
*No*	132 (68%)

## Discussion

The study demonstrated a significant association between a history of fall and preadmission medications taken. It highlighted complexities with medication management among older adults and identified reduced adherence to medication as a potential risk factor for falls.

Anticholinergic and sedative medication were identified in the study as fall-risk increasing drugs using measurement of the DBI and AEC. The DBI quantitatively evaluates exposure to anticholinergic or sedative medications and the AEC score quantifies anticholinergic medication use. In this study it was encouraging to see that increase in age was negatively associated with both the number of regular medications taken (*p* = 0.002) and anticholinergic or sedative use (*p* = 0.002 for DBI score ≥ 1 and *p* = 0.024 for AEC score of ≥ 2). Accordingly, all results were adjusted for age. Once adjusted for age, the results showed a positive association between having a DBI score ≥ 1 and a history of ≥ 1 falls (AOR = 2.2, 95% CI 1.0–4.8, *p* = 0.041). The increased risk of fall with high anticholinergic and sedative medication use demonstrated in this study is consistent with previous research [20–22, 38]. The pharmacological actions of these medications, such as decreased coordination and motor activity and increased risk of sedation, confusion, and blurred vision, are thought to contribute to the increased risk of falls. Anticholinergic medication use (indicated by the AEC scale) may confer the highest risk (AOR = 2.8, 95% CI 1.3–6.1, *p* = 0.010). In this study, 40 participants had a DBI score of ≥ 1, and 40 had an AEC ≥ 2. Both tools measure anticholinergic medication use. Therefore, 26 participants were recorded in both groups. However, 14 participants had an AEC score of ≥ 2 but had a DBI score of < 1. This highlights the fact that categorizing or measuring anticholinergic medication can vary among tools. For example, using the DBI score, the medication dose is considered, whereas using the AEC score, the medication dose is not. Further research is needed to establish the most effective method for identifying fall risk relating to anticholinergic and sedative use.

Antihypertensive use was not found to confer an increased risk of falls, with BB use being statistically more prevalent in non-fallers than in fallers. In the literature, there have been inconsistent findings for antihypertensives and related falls, and the associations are complicated by drug classes, duration of use, and patient factors [39]. The risk of antihypertensive-related falls may be highest within 30 days of initiation and less likely with long-term use [40]. In this study, the negative association between BB use and falls may have arisen because of differences between individuals rather than the effect of BBs. Nevertheless, previous research has indicated that chronic BB use may reduce the risk of falls [11] or that cardioselective BB use does not increase the risk of falls [41]. Furthermore, 85% of the BBs taken were cardioselective.

This study demonstrated that a high complexity of medication (indicated by an MRCI score ≥ 20) was significantly associated with a history of falls (AOR = 2.0, 95% CI 1.1–3.7, *p* = 0.018). This was more positively associated with a recent fall than just increasing the number of medications. The more complex regimens may probably result in reduced compliance and increased risks of medication misadventure. The MRCI scores each medication/medication type in terms of its difficulty of use, frequency of use, and whether there are additional instructions. It has been previously used to indicate patients at risk of nonadherence [33].

Adherence to and optimisation of medication management in the older population is challenging. The presence of age-related features/clinical symptoms, such as mild cognitive impairment, vision impairment and hearing loss were positively associated with reduced understanding of medications. In this study, 17% of the participants did not know why they were taking their medications, and 42% had an indicator of reduced compliance (either reporting that they forgot medication, ran out of medication, or intentionally missed medication). Furthermore, 86% of the participants with an indicator of reduced compliance reported that they did not have anyone to help them with managing their medications. Forgetting medications was significant for a history of falls (*p* = 0.043). The findings of this study support those of existing research. A previous study showed that older people with low adherence to medications may experience a 50% increased risk of falling compared with peers reporting high adherence [7]. Furthermore, a recent study proposed that gaps in antihypertensive adherence may be a marker of increased risk of falls [42]. Further studies are needed to investigate how to improve compliance and whether adherence-focused interventions can reduce falls. Regular medication reviews, avoiding prescription of cascades, and deprescription of inappropriate or unnecessary medications are essential for reducing polypharmacy. Additionally, other interventions, such as dose administration aids, have been reported to improve compliance [43]. In this study, of the 57 participants using 10 or more regular medications, 56% did not have their tablets blister packed by their pharmacy. Using combination tablets whenever possible may also simplify regimens and improve adherence [44].

It was also identified from the study that participants were prescribed multiple medications that may lead to an increased risk of injuries should a fall occur. Of note, over a quarter of the participants who fell were taking anticoagulant medications, and a third were on antiplatelet medications. Therefore, medication review should also continue to consider the appropriateness of these medications, as well as optimizing therapeutics for bone health.

As a secondary finding, the presence of hearing impairment was found to be significant with falls in this study. This supports the existing evidence that suggests hearing loss is an independent risk factor for falls [45, 46]. While the aetiology of hearing impairment on falls is likely multifactorial, this study found that hearing impairment was positively associated with self-reported difficulties with medication management.

This study has several limitations. First, the participants included in the study were not randomised but included a convenience sample of individuals interviewed by four independent researchers before discharge. The long study duration reflects the pauses required due to COVID-19 restrictions. Second, fall history was determined by self-report, which may have been influenced by the patient’s perspective of a “fall,” reduced cognition, and concerns about admitting falls at home that may affect their discharge. Therefore, medical notes for any documented falls in the past 6 months were reviewed to improve the accuracy of our results. However, not all falls may have been documented. Third, medication adherence was also determined by self-report using a study-specific questionnaire. Assessing medication adherence is an area that requires further research; It is noted that of the existing ‘validated’ tools available, none have been found to be superior [47]. Patients may have over-reported adherence, especially as they were in the hospital at the time of the interview. Finally, the DBI and AEC scores were measured only for regular medications. The effect of *pro re nata* medication use was not examined in this study. Although most medications were recorded in the lists used for DBI calculation [29] and AEC calculation [31], a very small number were not classified and could not be included in the DBI or AEC calculations.

## Conclusion

Older patients may be considered a vulnerable group at risk of falls. Medications such as sedatives and anticholinergic agents increase the risk of falls and should be avoided where possible. A decisive approach to simplifying complex medication regimens and employing strategies to help patients manage their medications correctly may contribute to a reduced risk of falls and subsequent morbidity. Further studies on other medications that may be associated with the risk of falls are needed.

## Supplementary Information

Below is the link to the electronic supplementary material.

Supplementary file1 (DOCX 363 KB)
