# Correction: Preadmission medications and recent falls in older inpatients: an observational study

**DOI:** 10.1007/s11096-025-01886-3

**Published:** 2025-03-08

**Authors:** Louise Clarkson, Anthony Griffiths, Shu‑Kay Ng, Alfred K. Lam, Tien K. Khoo

**Affiliations:** 1 School of Medicine and Dentistry, Grifth University, Gold Coast, QLD Australia; 2 Northern New South, Wales Local Health District, Lismore, NSW Australia; 3 Graduate School of Medicine, University of Wollongong, Wollongong, NSW Australia

**Correction to: International Journal of Clinical Pharmacy** 10.1007/s11096-024-01859-y

In Table 2 of this article, the data under the row “Antihypertensive Medication” and their values were aligned incorrectly.

For completeness and transparency, the old incorrect version and the corrected version of Table 2 are displayed below

The original article has been corrected.

Incorrect version of Table 2

**Table:** Table 2 Pharmacological treatment on admission

	All participants (*n* = 194)	Participants with no falls (*n* = 87, 44.8%)	Participants with one or more falls (*n* = 107, 55.2%)	*p*-value
DBI = 0	100 (51.5%)	50 (57.5%)	50 (46.7%)	*p* = 0.136
DBI ≥ 1	40 (20.6%)	14 (16.1%)	26 (24.3%)	*p* = 0.160
AEC ≥ 2	40 (20.6%)	12 (13.8%)	28 (26.2%)	***p*** ** = 0.034**
Antihypertensive Medication -Thiazides and related antihypertensives ACEI’s ARBS CCB’s BB’s Other antihypertensives	19 (9.8%) 51 (26.3%) 55 (28.4%) 43 (22.2%) 78 (40.2%) 16 (8.2%)	10 (11.5%) 26 (29.9%) 28 (32.2%) 21 (24.1%) 42 (48.3%) 8 (9.2%)	9 (8.4%) 25 (23.4%) 27 (25.2%) 22 (20.6%) 36 (33.6%) 8 (7.5%)	*p* = 0.472 *p* = 0.305 *p* = 0.285 *p* = 0.551 ***p* = 0.039** *p* = 0.665
Statin Use	104 (53.6%)	53 (60.9%)	51 (47.7%)	*p* = 0.066
Proton Pump Inhibitor Use	86 (44.3%)	36 (41.4%)	50 (46.7%)	*p* = 0.456
Anticoagulant Use	51 (26.3%)	23 (26.4%)	28 (26.2%)	*p* = 0.966
Antiplatelet Use	74 (38.1%)	38 (43.7%)	36 (33.6%)	*p* = 0.152

*ACEI* Angiotensin-Converting Enzyme Inhibitor, *ARB* Angiotensin Receptor Blocker, *CCB* Calcium Channel Blocker, *BB* Betablockers

The correct version of Table 2

**Table:** Table 2 Pharmacological treatment on admission

	All participants (*n* = 194)	Participants with no falls (*n* = 87, 44.8%)	Participants with one or more falls (*n* = 107, 55.2%)	*p*-value
DBI = 0	100 (51.5%)	50 (57.5%)	50 (46.7%)	*p* = 0.136
DBI ≥ 1	40 (20.6%)	14 (16.1%)	26 (24.3%)	*p* = 0.160
AEC ≥ 2	40 (20.6%)	12 (13.8%)	28 (26.2%)	***p*** ** = 0.034**
Antihypertensive Medication				
Thiazides and related antihypertensives	19 (9.8%)	10 (11.5%)	9 (8.4%)	*p* = 0.472
ACEI’s	51 (26.3%)	26 (29.9%)	25 (23.4%)	*p* = 0.305
ARBS	55 (28.4%)	28 (32.2%)	27 (25.2%)	*p* = 0.285
CCB’s	43 (22.2%)	21 (24.1%)	22 (20.6%)	*p* = 0.551
BB’s	78 (40.2%)	42 (48.3%)	36 (33.6%)	***p*** ** = 0.039**
Other antihypertensives	16 (8.2%)	8 (9.2%)	8 (7.5%)	*p* = 0.665
Statin Use	104 (53.6%)	53 (60.9%)	51 (47.7%)	*p* = 0.066
Proton Pump Inhibitor Use	86 (44.3%)	36 (41.4%)	50 (46.7%)	*p* = 0.456
Anticoagulant Use	51 (26.3%)	23 (26.4%)	28 (26.2%)	*p* = 0.966
Antiplatelet Use	74 (38.1%)	38 (43.7%)	36 (33.6%)	*p* = 0.152

*ACEI* Angiotensin-Converting Enzyme Inhibitor, *ARB* Angiotensin Receptor Blocker, *CCB* Calcium Channel Blocker, *BB* Betablockers

