# Supplementary material for: Preadmission medications and recent falls in older inpatients: an observational study
Source: Int J Clin Pharm. 2025 Feb 7;47(3):708–16. doi: 10.1007/s11096-024-01859-y (PMC12125044; doi:10.1007/s11096-024-01859-y)
Supplement: Supplementary file 1 — Supplementary file1 (DOCX 363 KB) [file 11096_2024_1859_MOESM1_ESM.docx]

## **Appendix 1** Patient Medication Questionnaire

| Q1. How many pills do you take each day? | 1–5 pills | 6–10 pills | 11–15 pills | 16–20 pills | More than 20 pills |
| --- | --- | --- | --- | --- | --- |
|  |  |  |  |  |  |
|  | 1× | 2× | 3× | 4× | 5× or more |
| Q2. How many times per day do you take medication? |  |  |  |  |  |
|  |  |  |  |  |  |
|  | 1 = Strongly Agree | 2 = Agree | 3 = Undecided | 4 = Disagree | 5 = Strongly Disagree |
| Q3. I know why I am taking each medication |  |  |  |  |  |
| Q4. I have no difficulties remembering to take my medication |  |  |  |  |  |
| Q5. At times, I intentionally do not take some of my medication |  |  |  |  |  |
| Q6. I never run out of medication |  |  |  |  |  |
| Q7. I find it easy to get my scripts filled for my medication |  |  |  |  |  |
| Q8. I am convinced of the importance of my medication |  |  |  |  |  |
| Q9. I worry that my prescription medication will do more harm than good |  |  |  |  |  |
| Q10. I feel financially burdened by my out of pocket expenses for my prescription medication |  |  |  |  |  |
|  |  |  |  |  |  |
|  | Yes | No |  |  |  |
| Q11. Do you ever forget to take your medication? |  |  |  |  |  |
|  |  |  |  |  |  |
|  | Yes | No |  |  |  |
| Q12. I independently take my medication |  |  |  |  |  |
| Q13. Someone else helps me take my medication |  |  |  |  |  |
| Q14. I use a Webster pack for my medication |  |  |  |  |  |
| Q15. I keep a list of medications I am currently taking |  |  |  |  |  |
|  |  |  |  |  |  |
|  | Yes | No |  |  |  |
| Q16. Do you have a hearing impairment? |  |  |  |  |  |
| Q17. Do people often have to repeat themselves when they talk to you? |  |  |  |  |  |
| Q18. Do you have problems with your vision? |  |  |  |  |  |
